# Interplay Between the Epigenome, the Microenvironment, and the Immune System in Neuroblastoma

**DOI:** 10.3390/cancers17111812

**Published:** 2025-05-29

**Authors:** Valentina Andrade-Perez, Noël J.-M. Raynal

**Affiliations:** 1 Département de Pharmacologie et Physiologie, Faculté de Médecine, Université de Montréal, Montréal, QC H3T 1C5, Canada; 2 Centre de Recherche de l’Hôpital Sainte-Justine, 3175, Chemin de la Côte-Sainte-Catherine, Montréal, QC H3T 1C5, Canada

**Keywords:** neuroblastoma, *MYCN*, epigenetics, chromatin, DNA methylation, tumor microenvironment, immunotherapy, natural killer cells

## Abstract

Neuroblastoma (NB) is the most prevalent extracranial childhood tumor and the third leading cause of death from cancer in children. Despite having a high overall survival rate for low- and intermediate-risk patients, survival rates for high-risk cases remain unsatisfactory. The current standard treatment for high-risk NB involves surgery, chemotherapy, radiotherapy, autologous stem cell transplantation, immunotherapy with anti-ganglioside GD2, and differentiation therapy with isotretinoin. Besides not being enough to achieve a high survival rate in high-risk patients, these treatments are associated with significant side effects. With next-generation sequencing technologies, a better understanding of the genetic and epigenetic landscapes of NB has been achieved. This has led to the study of novel treatments to improve the overall survival rate of high-risk NB and reduce the toxicity of conventional treatments. Current research is focusing on the development of targeted drugs for genetic and epigenetic alterations, and protein degraders. Moreover, immunotherapy to enhance anticancer immune responses and by using cell-engineering techniques with chimeric antigen receptor (CAR) T and NK cells are being explored to target NB cells. Here, we review promising novel treatment strategies for NB, which target genetics, epigenetics, the tumor microenvironment, and the immune landscape, highlighting preclinical studies and ongoing clinical trials.

## Simple Summary

Neuroblastoma (NB) remains a clinically challenging cancer with high heterogeneity and poor outcomes in high-risk patients. The aim of this review is to provide an integrated synthesis of the key mechanisms that drive NB progression, particularly oncogenes, epigenetic regulators, and the tumor microenvironment, and how they can be targeted with current therapeutic strategies, including immunotherapy. By examining how these mechanisms contribute to NB progression and treatment resistance, this review highlights opportunities for developing more effective and rationally designed combination therapies. Ultimately, this work seeks to propose future research directions that could improve therapeutic outcomes for children affected by this aggressive disease.

## 1. Introduction

**Figure 1:**
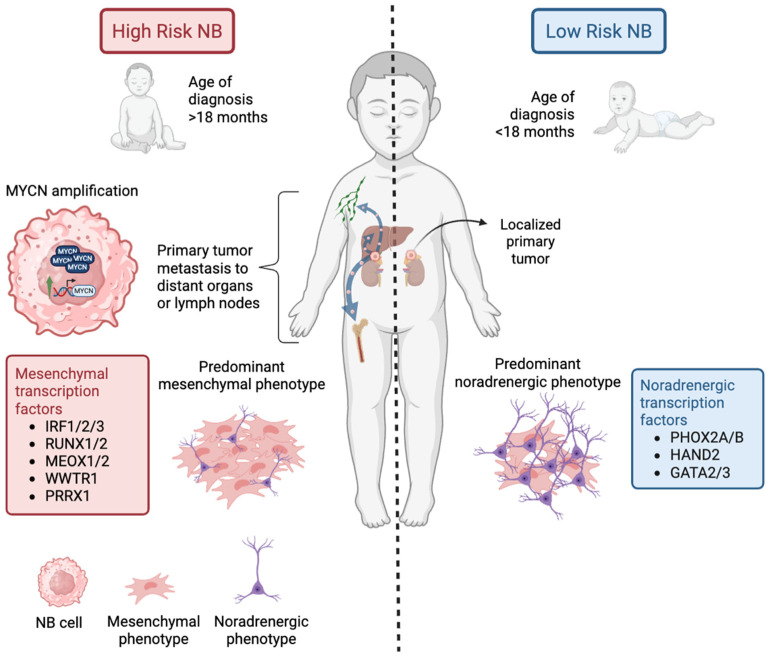
Differences between low-risk and high-risk NB. Primary NB tumors arise most commonly in adrenal glands or sympathetic ganglia. Low-risk NB has a high overall survival rate, and it is diagnosed in patients younger than 18 months, with tumors that are locoregional and primarily composed of noradrenergic cells. These low-risk tumors exhibit expression of the transcription factors *PHOX2A/B*, *HAND2*, and *GATA3*. In contrast, high-risk NB is associated with several aggravating factors like an age of diagnosis greater than 18 months, *MYCN* amplification, and metastasis of the primary tumor to distant sites such as the liver, cortical bones, and lymph nodes. Furthermore, the primary tumor cells in high-risk NB predominantly express the more invasive mesenchymal phenotype, characterized by the transcription factors *IRF1/2/3*, *RUNX1/2*, *MEOX1/2*, *WWTR1*, and *PRRX1*. Created with BioRender.com.

Neuroblastoma (NB) is a heterogeneous solid tumor of the sympathetic nervous system that usually arises in the adrenal gland and sympathetic ganglia [1,2]. It is the most common extracranial solid tumor in infants and accounts for 10% of deaths from cancer in the pediatric population, making it the third leading cause of morbidity among children with cancer [3,4]. It is usually diagnosed before the age of 10 and the age of diagnosis is inversely proportional to the chance of survival. Thus, a higher age of diagnosis is associated with a poorer prognosis [5,6]. Children younger than 18 months have an 88% overall survival rate, whereas after the age of 12, the survival rate drops below 10% [5]. As a result, age at diagnosis is one of the factors used to classify NB as low- or high-risk. Other factors used by the Children’s Oncology Group for this classification include stage, tumor histology, 11q status, *MYCN* amplification, and DNA ploidy [7]. This classification determines the clinical interventions for NB patients, which goes from low-risk cases that need observation or surgery, to high-risk cases that require intensive multi-modal therapy that includes chemotherapy, surgery, or myeloablative chemotherapy, followed by stem cell transplantation and immunotherapy with ganglioside 2 (GD2) antibodies [8,9]. The main differences between low-risk and high-risk NB are summarized in Figure 1.

The International Neuroblastoma Risk Group Staging System (INRGSS) classifies NB tumors based on the presence or absence of metastatic disease and of image-defined risk factors, which are features associated with increased surgical risk due to tumor location [10,11]. Tumors that are confined to one body compartment and lack image-defined risk factors are considered stage L1, whereas locoregional tumors that show one or more image-defined risk factors are classified as stage L2 [11,12]. Stage M indicates distant metastatic disease, except in cases classified as MS, which refers to metastatic NB confined to the skin, liver, and bone marrow without cortical bone involvement, in patients younger than 18 months [11,12].

Histologically, NB consists of small round cells named neuroblasts, which are composed of mesenchymal and differentiated noradrenergic cell populations [13,14]. NB may originate from early migratory neural crest cells or late Schwann cell precursors undergoing an impaired differentiation into sympathetic neurons due to epigenetic and genetic defects [15]. NB tumors could appear at any location where these types of cells are found [13,15]. In the human adrenal gland, the differentiation of Schwann cell precursors into sympathoadrenal states takes several weeks. This long duration of the differentiation process, along with its complexity and plasticity, allow NB emergence in this anatomical compartment during child development [16]. Based on the differentiation state of the cells, they can be classified as follows: undifferentiated, poorly differentiated, and differentiated. Depending on the age of diagnosis, these histology groups can be classified as favorable or unfavorable, with the latter being constituted by the undifferentiated state at all ages, poorly differentiated at ages older than 1.5 years, and differentiated at ages older than 5 years [9]. Tumors with an unfavorable histology are associated with poor clinical outcomes and resistance to the current therapeutic regimens. Considering the effects of tumor histology on prognosis, one potential approach for novel therapies is to promote differentiation to improve clinical outcomes.

According to the Children’s Oncology Group (COG)’s risk classifier of 2021, low-risk NB patients of all ages include those with INRGSS L1 tumors. Additionally, patients younger than 18 months who are classified as having stage MS disease and whose tumor does not have *MYCN* amplification with all favorable features (histology, diploidy, or segmental chromosomal aberrations) are also classified as low risk [17]. These patients have an expected overall survival (OS) rate greater than 95% [17]. The treatment for low-risk cases begins with observation, as some tumors will spontaneously regress without therapy, or complete surgical resection when possible [17,18]. In contrast, intermediate-risk patients who undergo moderate intensity therapy exhibit high OS between 85 and 90%. Their treatments include low-intensity chemotherapy and surgical resection [17,19]. These patients lack *MYCN* amplification and are at stages L2, MS with one unfavorable feature, and stage M in children younger than 18 months [17,19].

High-risk NB patients have poor outcomes, with 5-year survival rates below 50% [4]. According to the COG, high-risk cases include children with stage M tumors diagnosed after 18 months of age and patients that have stage L2, MS, or M tumors with *MYCN* amplification [17]. These cases have an initial 18-month treatment scheme that consists of three phases [20]. The induction phase aims to achieve remission using multi-agent chemotherapy and surgical resection of the tumor [20,21]. However, only 20% of patients show a complete response at the end of this phase [21]. The goal of the consolidation phase is to eliminate any remaining cancer cells through intensive chemotherapy with autologous stem cell transplantation followed by radiotherapy [20,21]. Finally, the post-consolidation treatment involves immunotherapy with ganglioside GD2 antibodies, which is an abundant glycolipid on the surface of NB cells, and differentiation therapy with isotretinoin, a known differentiating agent [20,21].

The composition of NB is greatly marked by the heterogeneity of its cell of origin, which complexifies the characterization of actionable targets. Moreover, NB cells express various transcription factors assembled into core regulatory circuitries (CRCs), which drive at least three main gene expression programs referred to as neuroblastic (N-type), flat or substrate adherent (S-type), and intermediate (I-type) [22]. In addition, NB phenotypes are also classified as noradrenergic and mesenchymal states. The CRC that drives a noradrenergic phenotype includes *PHOX2A/B*, *HAND2*, and *GATA2/3* transcription factors. Other transcription factors have been associated with mesenchymal states, such as *IRF1/2/3*, *RUNX1/2*, *MEOX1/2*, *WWTR1*, and *PRRX1* [13,23,24]. Each NB phenotype is characterized by specific invasion/migration capabilities and drug responses. The noradrenergic state is associated with low-risk NB, and it shows a higher degree of differentiation. This aligns with the expression of *PHOX2B*, *HAND2*, and *GATA3*, known to be involved in the differentiation of the noradrenergic sympathetic nervous system [25]. In contrast, the mesenchymal phenotype is highly expressed in high-risk NB and exhibits enhanced migratory and invasive properties and drug resistance to cisplatin and doxorubicin [2,25]. Although it has not been fully established whether mesenchymal cells are directly responsible for metastasis in NB, there is enriched mesenchymal gene expression in distant metastases and poor prognosis tumors [26,27]. The migratory capacity of this phenotype is mainly regulated by the NOTCH3 pathway, suggesting a potential therapeutic target to limit metastatic spread [26]. Moreover, high-risk NB presents a high expression of an intermediate cell type between noradrenergic and mesenchymal states. This intermediate state shows stem-like characteristics and may further contribute to the heterogeneity of high-risk NB [25].

Advanced single-cell approaches have provided new insights into the genetic and epigenetic landscape of NB, increasing the understanding of how intratumoral heterogeneity drives therapeutic resistance. For instance, the single-cell RNA sequencing of chemo-resistant NB cells showed several subpopulations with altered cell identities and dynamic plasticity between noradrenergic and mesenchymal phenotypes, indicating a mesenchymal transition during chemotherapy but a predominant noradrenergic state in relapse after chemotherapy [28].

Therefore, the mesenchymal phenotype also plays a crucial role in therapeutic resistance, as demonstrated by its enrichment after chemotherapy and in relapse tumors [23]. Through transcriptional and epigenetic reprogramming, resembling the epithelial-to-mesenchymal (EMT)-like processes documented for several other tumors, noradrenergic cells can acquire mesenchymal features in response to stress, further contributing to therapeutic resistance [27]. Importantly, this acquired resistance may also drive tumor progression by inducing the expansion of aggressive, therapy-adapted cell populations that survive treatment. Considering the distinct phenotypes and their correlation with the aggressiveness of NB, there is potential to leverage this plasticity between these cell types to enhance the therapeutic response. To address this, rational drug combinations that simultaneously target both the noradrenergic and mesenchymal populations or strategies to block transitions between these phenotypes can be explored. For instance, the pharmacological inhibition of the noradrenergic to mesenchymal transition, mainly through epigenetic drugs, has been reviewed as a potential strategy to prevent therapy-induced shifts toward a resistant mesenchymal phenotype [29].

Currently, NB treatments still produce a severe burden for patients due to significant short-term and long-term side effects, especially for high-risk NB cases. Radiotherapy can cause growth hormone deficiency, skeletal dysplasia, diabetes mellitus, cardiac/pulmonary/renal dysfunctions, and subsequent malignancies [30]. For example, chemotherapeutic drugs like cisplatin and carboplatin have been associated with hearing loss and renal dysfunction. Busulfan treatment can result in pulmonary dysfunction. The use of anthracyclines is linked to cardiac dysfunction and secondary malignancies [30]. Five years after the completion of therapy, 38% of NB patients presented endocrine issues related to growth and thyroid dysfunction, 49% suffered dental complications, 72% had hearing impairment, and 46% showed neurocognitive deficits [31]. These findings highlight the urgent need for new therapeutic approaches to reduce the burden of late effects in survivors of NB.

In this review, we present an overview of the latest advancements in the understanding of NB tumor biology and treatment approaches. We will discuss oncogenetic and epigenetic aberrations of NB, and therapies focused on these aspects. Moreover, we will explore the tumor microenvironment (TME) and the loss of immune function, immunotherapy, and the promising role of NK cells in the treatment of NB. This review brings a unique perspective by integrating oncogenic, epigenetic, and TME drivers of NB progression, which are mechanisms often discussed in isolation, and critically examining how they shape resistance and response to immunotherapy. By highlighting emerging preclinical strategies and clinical trials, it offers a guide for rational combination therapies with the potential to improve NB outcomes.

## 2. Oncogenes in Neuroblastoma

Tumor genetics is another important aspect to classify the clinical outcomes of NB. Specifically, the NB genotype can show segmental chromosomal alterations that are associated with poor prognosis. These include deletions on segments that carry tumor suppressor genes such as 1p, 6q, and 11q, or gains on segments that contain oncogenes such as 1q, 2p, and 17q [32]. The main genetic alterations related to poor prognosis in NB include *MYCN* amplification and *ALK* mutations [15].

### 2.1. MYCN

The *MYC* genes consist of a family of three proto-oncogenes: *MYCN*, *MYC*, and *MYCL*, encoding for transcription factors with a basic helix-loop-helix structure [33]. *MYCN* is involved in normal neural development as it regulates the expression of target genes that maintain pluripotency and is involved in the proliferation and growth of developing nervous system cells [33,34]. Specifically, *MYCN* downregulation reduces cell cycle progression and arrests human neural crest stem cells in the G0/G1 phase, highlighting its essential role in NB cell proliferation [35]. Thus, when *MYCN* is overexpressed, it leads to NB formation [5].

*MYCN* amplification is detected in 20–30% of NB patients and is present in approximately 50% of high-risk cases [36]. MYCN overexpression is attributed not only to *MYCN* genetic amplification, but is also caused by transcriptional activation, increased protein stabilization, or decreased degradation [36]. As previously mentioned, *MYCN* is crucial in the regulation of the cell cycle. Its overexpression alters normal cell cycle progression, causing apoptosis inhibition and induced proliferation [37,38]. Moreover, it generates metabolic changes that result in increased nutrient absorption, macromolecule synthesis, and energy production, promoting cell growth and proliferation [39]. Additionally, *MYCN* upregulates the expression of pluripotency-associated genes in NB cells, including *LIF*, *KLF2*, *KLF4*, *LIN28B,* and *TWIST1* [40,41]. Overall, *MYCN* overexpression disrupts the normal cell cycle and promotes gene expression associated with stemness, which confers stem cell-like characteristics and promotes metastasis in NB [36].

Due to its role in NB, *MYCN* is a promising therapeutic target for *MYCN*-amplified cases. However, the direct targeting of MYCN is limited by the lack of catalytic site where small molecule inhibitors could bind. Therefore, indirect approaches targeting MYCN protein stability are currently under research [42]. Such strategies aim to selectively impair the survival and proliferation of MYCN-amplified tumor cells, potentially offering more personalized treatment options for high-risk patients. Examples of these approaches include the inhibition of aurora kinase A (AURKA), which stabilizes MYCN and prevents its proteasomal degradation [42]. Alisertib, a known AURKA inhibitor, has been studied for the treatment of NB. Despite showing an effective antitumor response in preclinical studies, its efficacy in patients was limited by toxicity when used in monotherapy and in combination with cytotoxic agents [43,44]. New AURKA inhibitors, such as CD532 and PHA-680626, have shown a greater effect than alisertib on MYCN protein stability, and decreased the viability of NB cells in xenograft models, but they remain to be studied in NB patients [45,46].

Another therapeutic approach to target MYCN indirectly is the use of bromodomain and extra-terminal (BET) inhibitors, mainly BRD4 inhibitors. BRD4 is a histone acetylation reader, which transmits the active epigenetic signal by recruiting MYCN to promoter regions. Thus, blocking the interaction between acetylated histones and BRD4 reduces MYCN’s oncogenic effects [47]. JQ1 is a well-characterized BRD2-3-4 inhibitor that competitively blocks the acetyl-lysine recognition pocket, thus removing BRD4 from chromatin [47]. JQ1 produced cytotoxic effects against NB cells in xenograft models, conferring prolonged survival but not complete regression [47]. This limited antitumoral effect can be explained by active kinome reprogramming, where cancer cells activate alternative pro-survival kinase pathways that counteract the effects of JQ1 [48]. I-BET151, another BRD4 inhibitor with a similar mechanism of action to JQ1, has been tested in combination with alisertib [49]. The alisertib and I-BET151 combination produced a synergistic interaction, driven in part by the role of I-BET151 to mitigate the upregulation of AURKA, MYC, and MYCN induced by AURKA inhibition. In four xenograft NB models, the combination improved overall survival compared to monotherapy. The efficacy of the combination was also augmented by the addition of the antimicrotubule drug vincristine [50].

An alternative approach against MYCN-driven NB to overcome the resistance of BET inhibitors is the use of PROTACs (proteolysis-targeting chimeras), which are bifunctional molecules that mediate selective proteasome degradation. Unlike traditional small molecule inhibitors that simply block protein function, PROTACs eliminate the target protein directly and can catalyze the simultaneous degradation of multiple copies, which enables lower dosing requirements and potentially decreases toxicity. In addition, they can overcome resistance mechanisms driven by protein mutations or upregulation [51]. PROTACs targeting BET (dBET57 and MZ1) have been shown to impair tumor growth in NB xenograft models, but further studies need to determine their clinical efficacy [52,53].

Targeting the metabolic pathways associated with *MYCN* amplification offers another promising approach in treating NB. For instance, *MYCN*-amplified tumors exhibit increased polyamine biosynthesis, which enhances cancer cell proliferation. This occurs because *MYCN* regulates ornithine decarboxylase (ODC), a key enzyme in polyamine synthesis, whose activity is increased in *MYCN*-amplified NB [54]. Thus, ODC inhibition reduces tumor growth and potentially improves patient outcomes. In 2023, the Food and Drug Administration (FDA) approved eflornithine, an irreversible inhibitor of ODC, to prevent relapse in high-risk NB [55]. Alternatively, depleting cysteine level may impact NB, because this amino acid is in high demand in *MYCN*-amplified NB. Indeed, reducing cysteine uptake triggers ferroptosis, resulting in tumor remission in an orthotopic *MYCN*-amplified NB model [56]. Moreover, in another study, it was found that the inhibition of fatty acid transport protein 2 (FATP2) resulted in the suppression of *MYCN*-induced glycerolipid accumulation and tumor growth in preclinical NB models [57].

It is worth nothing that there have also been attempts to target *MYCN* directly using a gene-level inhibitory molecule with a sequence-specific gene regulator. By generating a *MYCN*-targeting pyrrole-imidazole polyamide (PIP), researchers developed MYCN-A3, which directly binds within the *MYCN* transcript and alkylates DNA at homing motifs. This approach reduced *MYCN* copy numbers in *MYCN*-amplified NB. MYCN-A3 suppressed tumor progression without toxicities, resulting in prolonged overall survival in *MYCN*-amplified NB [58]. In addition, a recent study showed that the development of a novel ^191^Pt-labeled PIP had cytotoxic activity against *MYCN*-amplified NB cells in vitro. However, biodistribution studies on xenograft mice showed the accumulation of the ^191^Pt-labeled PIP in off-target sites [59]. Thus, despite the various strategies to target *MYCN*, a successful approach remains to be discovered.

### 2.2. ALK

Anaplastic lymphoma kinase (ALK) is a receptor tyrosine kinase that is highly expressed in the embryonic nervous system, while its expression is downregulated after birth [32,60]. ALK has been detected in mice models during neural crest delamination and migration, suggesting its role in the development of the nervous system [61]. *ALK* constitutes the most common single-gene mutation in primary NB, being present in 8–10% of all NB cases at diagnosis, reaching 17% in relapsed NB [61,62,63]. Hereditary NB is primarily caused by germline mutations in *ALK* [64]. Mutations in this gene are involved in NB oncogenesis, and they are associated with poor clinical outcomes in high- and intermediate-risk patients [32,60,62]. Consequently, ALK is a key target in NB.

Targeting ALK is mainly achieved using small molecule inhibitors to suppress its activity. Crizotinib is an ATP-competitive inhibitor of ALK and Met tyrosine kinases, approved for lung cancer and lymphoma that are *ALK*-positive. Crizotinib shows antitumor activity against NB in preclinical models, except for those containing the F1174L-mutated *ALK* (most common mutation in NB) that are resistant to the drug [65]. In phase II clinical trials, a 15% objective response rate was achieved in NB patients, being effective only in patients carrying the somatic *ALK* R1275Q mutation [66]. Lorlatinib, another ALK inhibitor that also inhibits ROS1 (a receptor tyrosine kinase), was approved in 2021 by the FDA for metastatic *ALK*-positive non-small cell lung cancer. Lorlatinib demonstrated a higher antitumor activity than crizotinib in preclinical studies, by being effective also in crizotinib-resistant NB models [67]. Lorlatinib exhibited safety and tolerability in a phase I trial against relapsed or refractory *ALK* mutation or *ALK*-amplified NB patients, which paves the way for further clinical trials [68]. Another approach to overcoming ALK inhibitor resistance is to directly target the *ALK* gene. Ota et al. developed a novel PIP conjugated with a DNA alkylating agent targeting the F1174L-mutated *ALK* gene, which proved to be cytotoxic against NB cells and showed antitumor activity in mice models [69]. The success of this approach underscores the potential of directly targeting the *ALK* gene as a viable strategy for overcoming the side effects of ALK inhibitors, and opens new avenues to improve outcomes in *ALK*-driven NB.

Besides these treatment approaches, CRISPR-Cas9 technology has become a powerful tool in NB research to underscore the genetic mechanisms of drug resistance and identify novel therapeutic combinations. High-throughput CRISPR/Cas9 knockout screens in *ALK*-positive NB cells have revealed genes whose loss reduces sensitivity to ALK tyrosine kinase inhibitors, providing insight into resistance pathways [70]. In addition, large-scale CRISPR screens in drug-treated cells have mapped genetic interactions that sensitize NB cells to standard chemotherapeutics. This approach showed that the inhibition of *PRKDC*, a key factor in the DNA damage repair machinery, could increase the effects of doxorubicin in high-risk NB cells [71]. Together, these studies highlight the potential of CRISPR screening in elucidating oncogenic vulnerabilities and optimizing treatment in NB.

## 3. Epigenetic Alterations in Neuroblastoma

**Figure 2:**
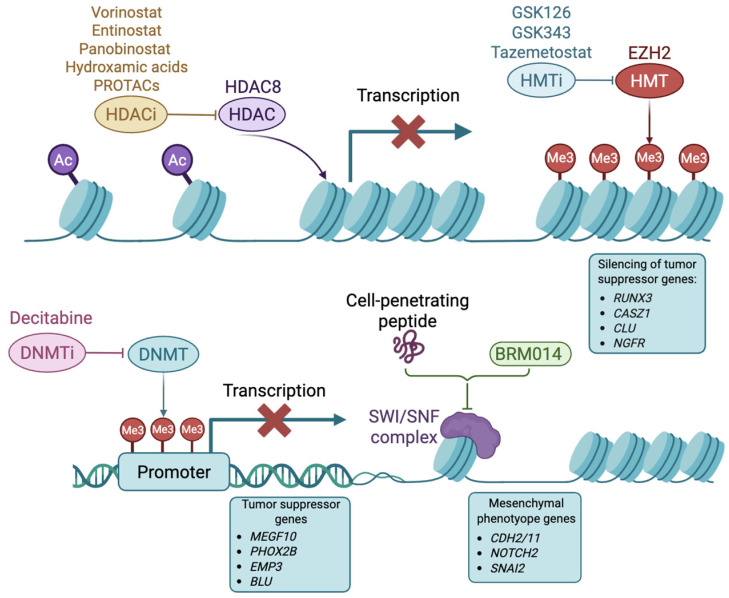
Epigenetic alterations in NB and treatment approaches. HDACs remove acetyl groups from histones, repressing gene transcription. In NB, HDAC8 is associated with poor outcomes, and current treatments focus on inhibiting its activity alongside other class I HDACs. Investigated HDAC inhibitors include vorinostat, entinostat, panobinostat, hydroxamic acids, and novel PROTACs. HMTs transfer methyl groups to histones, impacting chromatin condensation. In NB, the HMT EZH2 silences tumor suppressor genes like *RUNX3*, *CASZ1*, *CLU*, and *NGFR*. Inhibitors like GSK126, GSK343, and FDA-approved tazemetostat target EZH2. DNA hypermethylation blocks transcription of tumor suppressor genes such as *MEGF10*, *PHOX2B*, *EMP3*, and *BLU*, with decitabine studied as a potential treatment. Additionally, the SWI/SNF complex drives mesenchymal phenotype gene expression (*CDH2/11*, *NOTCH2*, and *SNAI2*), with ongoing research exploring inhibitors like BRM014 and cell-penetrating peptides. Created with BioRender.com.

There has been a growing focus on targeting the NB epigenome, driven by a better understanding of epigenetics’ influence on gene expression. Alterations in the epigenome can drive tumorigenesis, as they disturb transcriptional patterns by silencing tumor suppressor genes and increasing oncogene expression [72]. Epigenetic changes play critical roles in NB and involve aberrations in DNA methylation patterns, histone modifications (acetylation and methylation), chromatin remodeling, and non-coding RNAs [73]. Epigenomic changes have been associated with clinical outcomes. In particular, DNA hypermethylation on CpG sites correlates with high-risk NB [74,75]. Figure 2 highlights the epigenetic mechanisms that drive NB tumorigenesis and the drugs targeting these mechanisms.

### 3.1. Histone Deacetylases

Histone deacetylases (HDACs) are enzymes that catalyze the removal of acetyl groups from lysine residues of histone and non-histone proteins. The loss of histone acetylation leads to a more compact chromatin, reducing gene expression. Among the family of HDACs, HDAC8 (a class I HDAC) plays key roles in NB, as its expression has been associated with enhanced tumor progression, metastasis, and poor clinical outcomes [76,77,78]. Consequently, the inhibition of HDAC8 has been explored as a promising therapeutic strategy. Vorinostat (suberoylanilide hydroxamic acid), a broad-spectrum HDAC inhibitor approved by the FDA for the treatment of refractory cutaneous Tcell lymphoma, did not show antitumor activity against NB when tested as a single agent in xenografts at clinical doses [79]. Recent studies have tested vorinostat in combination with other agents to elicit a more effective antitumor response. The combination of vorinostat with SE486-11, a novel pyridobenzimidazole compound, had a synergistic interaction, inhibiting tumor growth in NB xenografts and in *MYCN* transgenic zebrafish and mice models. Interestingly, the combination of vorinostat and SE486-11 increased MYCN ubiquitination and degradation by reducing ubiquitin-specific protease 5 (USP5) levels. This study reveals that USP5 acts as an oncogenic cofactor in *MYCN*-driven NB and suggests that the combination of vorinostat and SE486-11 has some therapeutic potential [80]. Another combination of vorinostat and PENAO (4-(N-(S-penicillaminylacetyl)amino)-phenylarsonous acid), a mitochondrial adenine nucleotide translocase 2 (SLC25A5/ANT2) inhibitor, resulted in synergistic reduced cell viability, and the induction of apoptosis in NB cells. In a mouse model, the combination delayed tumor progression [81]. The pan-HDAC inhibitor panobinostat has been tested in combination with CBL0137, a compound that inhibits the histone chaperon FACT (Facilitates Chromatin Transcription) [82]. CBL0137 alone produced cytotoxic activity against NB in vitro, decreased tumor growth, and hindered NB tumorigenesis in xenograft models [83,84]. When used in combination with panobinostat, it produced stronger activity against high-risk NB mice models [82].

Since broad-spectrum HDAC inhibitors lack selectivity, their clinical use is associated with adverse effects like nausea, fatigue, diarrhea, anorexia, myelosuppression, and cardiac toxicity [85]. To overcome these challenges, researchers have developed more selective inhibitors against cancer-specific HDACs. Against NB, small molecule inhibitors with HDAC8 specificity (such as 1-naphthohydroxamic acid, PCI-34051, and 3-benzylamino-benzhydroxamic acid) demonstrated potent effects in vitro and in mice models, causing the induction of cell differentiation and reduction in tumor growth [86,87,88,89]. These specific small molecule inhibitors block the activity of HDACs by binding to their active site [90]. Alternatively, PROTACs were developed to target HDAC8 specifically, resulting in HDAC8 ubiquitination and subsequent proteasomal degradation. The evaluated compounds induced effective anti-NB activity in vitro [91]. However, they remain to be tested in vivo to confirm their preclinical potential as a therapeutic approach for NB.

Transcriptional reprogramming or therapy resistance induces the mesenchymal state of NB, which is more immunogenic with ligand expression and inflammatory cytokine secretion. Thus, the mesenchymal state promotes T cell and NK cell cytotoxicity and responds to immune checkpoint blockade [92]. This research underscores the potential of HDAC inhibitors in enhancing immunogenicity and immunotherapy, which could potentially improve therapeutic outcomes in NB. Therefore, the use of HDAC inhibitors demonstrated beneficial outcomes, particularly combining them with other agents in order to achieve an effective therapeutic response, although further research is needed to test the potential of these approaches in clinical settings.

### 3.2. Histone Methyltransferases

Histone methyltransferases had a methyl group on specific amino acid residues (such as lysine, arginine, and histidine) on histone tails. Depending on the specific histone residue that is methylated and the extent of methylation (mono-, di-, or tri-methylation), histone methylation can lead to either the activation or repression of gene expression. For instance, EZH2 is a histone methyltransferase overexpressed in NB that catalyzes the mono-, di-, and tri-methylation of lysine 27 on histone H3 (H3K27), repressing gene expression [72]. EZH2 helps to maintain the undifferentiated phenotype of NB by silencing several genes including *RUNX3*, *CASZ1*, *CLU*, and *NGFR* [93]. Mellini et al. reported that GSK126, a small molecule inhibitor of EZH2, reduced NB cell proliferation [94]. Bownes et al. investigated the antitumor potential of another EZH2 inhibitor, GSK343, showing its ability to diminish NB cell viability, promote differentiation, and suppress tumor growth in vivo [95]. Further studies need to be carried out to determine its clinical efficacy in NB.

### 3.3. DNA Methyltransferases

Another epigenetic mechanism that is altered in NB is DNA methylation. It is mediated by DNA methyltransferases that add methyl groups to cytosine residues within CpG sites. Cytosine methylation in promoter regions reduces gene expression. As reported by Ushijima et al., DNA hypermethylation in promoter regions is associated with poor prognosis in NB [96]. Localized DNA hypermethylation has been involved in the gene silencing of tumor suppressor genes like PHOX2B, MEGF10, EMP3, and BLU, whose downregulation promotes an undifferentiated state and increases NB cell proliferation and migration [97]. Interestingly, hypermethylation in the promoter region of TERT has been associated with dysregulated telomerase expression, enhancing tumor progression [98]. Thus, there is a rationale to target DNA hypermethylation in NB.

One of the most studied DNA methyltransferase inhibitors for the treatment of NB is decitabine (5-aza-2′-deoxycytidine), which decreases NB cell proliferation and induces differentiation [99]. However, it was reported in a phase I study in children with refractory NB that the dose of decitabine required to achieve antitumor effects exceeded the tolerated clinical dose [100]. This dose-limiting toxicity, in addition to rapid plasma clearance, constitutes a major challenge in achieving clinically significant effects with decitabine. Therefore, studies now focus on combining it with other agents to lower the dose needed to achieve antitumor effects. Hattori et al. performed preclinical studies to determine the effect of the combination of tamibarotene, a synthetic retinoic acid, with decitabine in NB [101]. As decitabine is known to promote the differentiating effects of all-trans retinoic acid (ATRA) and 13-cis-retinoic acid, the authors hypothesized that it could enhance the effect of tamibarotene. Indeed, pre-treatment with decitabine followed by tamibarotene increased the expression of differentiation markers (ARHGEF3, HOXD4, GAP43, and NTRK1) and inhibited tumor growth in xenograft models with moderate adverse effects, supporting further investigations into this combination [101]. Moreover, a recent study confirmed the antitumor effect of DNA methyltransferase 1 inhibition in NB, which decreased tumor growth and sensitized the cells to chemotherapy in preclinical models [102]. Despite these promising results, the broader applicability of DNA methyltransferase inhibitors in the clinic, like other epigenetic drugs, is limited, which leads to variable treatment responses across patients, and the careful selection of compatible combinations to optimize the antitumor effect while minimizing side effects.

### 3.4. Chromatin Remodelers

Chromatin remodeling is an epigenetic process that catalyzes the modification of the chromatin structure to regulate its degree of condensation [73]. The Switching defective/Sucrose Non-Fermenting (SWI/SNF) complex destabilizes histone–DNA interactions, enabling transcriptional activation and elongation [73]. Silencing BRG1-associated factor (BAF), a subtype of SWI/SNF, leads to the epigenetic repression of genes related to a mesenchymal phenotype, including *CDH2/11*, *NOTCH2*, and *SNAI2*, which reduces cell-invasive properties and inhibits NB metastasis [103]. Hu et al. developed a cell-penetrating peptide to block the interaction between cellular nucleic acid-binding protein (CNBP, a regulator of SWI/SNF) and the SMARCC2 protein. The peptide inhibited NB progression in mice [104]. Recently, Cermakova et al. showed that using the small molecule inhibitor BRM014 to inactivate SWI/SNF ATPase activity arrests NB cells in G1 and sensitizes NB cells to retinoid-induced differentiation [105]. Despite significant advances, uncovering the therapeutic potential of SWI/SNF in NB treatment requires further investigation to translate these findings into pharmacological interventions suitable for clinical application.

### 3.5. Super-Enhancers

Super-enhancers are epigenetic regulatory elements, constituting a region of large transcriptional enhancer clusters that play an important role in the increased expression of oncogenes in cancer. Particularly in NB, super-enhancer regions are crucial in regulating noradrenergic and mesenchymal states and determining cell and lineage identity. Super-enhancers mediate the expression of noradrenergic enzymes like dopamine-β-hydroxylase and tyrosine hydroxylase, as well as adrenergic/noradrenergic markers such as PHOX2B, enolase, and neurofilament proteins [25]. Furthermore, they also mediate the expression of mesenchymal markers, including vimentin, fibronectin, β2-microglobulin, and HLA class 1 antigens [25].

Recent work has shown that super-enhancers may also determine therapeutic responses. A study investigating ATRA-mediated differentiation in NB showed that noradrenergic and mesenchymal states have distinct super-enhancer landscapes, correlating with their ability to undergo or resist differentiation upon treatment [106]. Particularly, this underscored a novel CRC induced by ATRA treatment that resulted in the upregulation of the super-enhancer-driven transcription factors *HIC1*, *SMAD3*, *RARA*, and *RARB*. In a recent study, ATRA treatment induced the downregulation of super-enhancer regions associated with *MYCN* and *SOX11*, followed by the upregulation of differentiation-related transcription factors like *SOX4* [107]. The functional silencing of *SOX11* using CRISPR interference (dCas9-KRAB) inhibited cell growth and reduced tumor volume in NB, whereas the silencing of *SOX4* confirmed its role in ATRA-induced differentiation. Another study further supported the importance of *SOX4* in ATRA-mediated effects, showing that its knockdown partially reversed these effects on proliferation and differentiation [108]. Moreover, this study showed that *SOX4* expression was positively correlated with survival in NB patients. Thus, targeting the epigenome to reprogram super-enhancers that regulate noradrenergic and mesenchymal states could offer a promising therapeutic approach to promote differentiation in NB.

Treatment with THZ1, a covalent CDK7 inhibitor, resulted in cytotoxic activity against *MYCN*-amplified NB cells and impaired tumor growth in xenograft mouse models, as it selectively impairs global transcription in these cells by targeting super-enhancer-associated genes related to the malignant state, such as *MYCN* and *ALK* [109]. Recently, the authors showed that the inhibition of CDK7-mediated transcription, through a more selective covalent inhibitor YKL-5-124, had synergistic effects when used in combination with JQ1 (BRD4 inhibitor), leading to reduced levels of *MYCN* expression and *MYCN*-associated oncogenes, the reduced proliferation of NB cells, and limited tumor growth in mice [110]. These findings highlight the therapeutic potential of combinatorial epigenetic approaches to target super-enhancer-driven transcriptional programs in high-risk NB. However, translating these findings into clinical practice remains challenging, due to intratumoral heterogeneity and the need for robust biomarkers that help identify patients whose tumors show dependency on super-enhancer-driven transcriptional programs.

## 4. Microenvironment in Neuroblastoma

**Figure 3:**
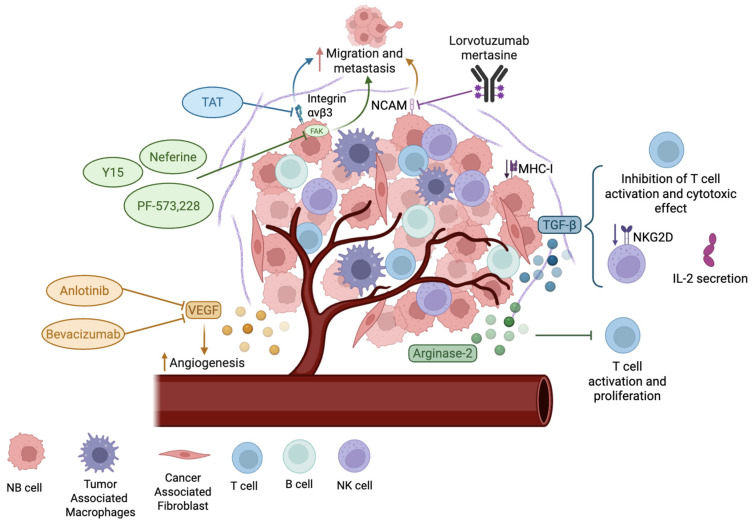
NB tumor microenvironment and treatments. The tumor microenvironment in NB consists of various cell types, including macrophages, cancer-associated fibroblasts, T cells, B cells, and NK cells. Several factors in the tumor microenvironment contribute to the immunosuppressive state of NB. Molecules like FAK, integrin αvβ3, and NCAM facilitate interactions between NB cells and the extracellular matrix (ECM), enhancing motility and invasiveness. Inhibitors targeting these molecules, such as Neferine, PF-573228, Y15 (FAK inhibitors), TAT (integrin αvβ3 inhibitor), and lorvotuzumab mertansine (anti-NCAM antibody-drug), are under study as treatments. Another approach is to target the proangiogenic molecule VEGF with the following inhibitors: anlotinib and bevacizumab. Furthermore, NB cells secrete immunosuppressive molecules like TGF-β, which inhibits T cell activation and cytotoxic effects, reduces NKG2D expression on NK cells, and promotes the secretion of IL-2. Arginase-2 is another immunosuppressive factor that blocks T cell proliferation and activation. MYCN overexpression also supports this immunosuppressive state by downregulating MHC-I expression on NB cells. Created with BioRender.com.

The tumor microenvironment (TME) is a complex and heterogeneous ecosystem where malignant cells and non-malignant cells interact with each other. Figure 3 highlights the features of the NB TME and potential treatments that target them. Besides tumor cells, the TME contains T cells, B cells, NK cells, cancer-associated fibroblasts, tumor-associated macrophages, endothelial cells, mesenchymal stromal cells, and Schwann cells, among others. The dynamic interaction between TME components leads to physical changes in the extracellular matrix (ECM), vascular remodeling, inflammation, and metastasis, thus contributing to tumor progression and heterogeneity [111]. The ECM is a network of extracellular molecules’ structural support and allows for the transmission of extracellular signals. Its main components include collagen, fibronectin, vitronectin, laminin, proteoglycans, and glycoproteins. In NB, changes in the ECM composition can influence morphology, biological functions, invasion phenotypes, inflammation, and immunosuppression, impacting tumor progression and cellular behavior [111,112,113]. In NB, a rigid ECM suppresses *MYCN* expression and reduces angiogenesis, as it downregulates the expression of vascular endothelial growth factor (VEGF) [114,115]. ECM stiffness can be induced by the presence of collagen I and III fibers, low levels of glycosaminoglycans, and high levels of vitronectin and fibronectin [116,117,118]. Moreover, ECM stiffness promotes chemoresistance through several signaling pathways, such as the binding of the matrix to integrin receptors, that lead to p53 degradation and NF-κB nuclear translocation, reduced apoptosis, and the upregulation of cancer stem cell-like markers. It also promotes genomic instability, glucose uptake, protein synthesis, and epithelial-to-mesenchymal transition, conferring a pro-metastatic and drug-resistant phenotype [113,119]. In addition, a stiff ECM constitutes a physical barrier that impairs effective drug delivery and promotes drug clearance in the TME [119,120]. Altogether, these adaptations allow cancer cells to evade drug treatments and sustain tumor progression.

Therefore, targeting the ECM’s interactions with cancer cells could modulate NB characteristics and improve therapeutic responses. One approach to this focuses on the focal adhesion kinase (FAK), a tyrosine kinase that regulates cellular adhesion and mediates the integrin signaling pathways stimulated by ECM stiffness [119]. In NB cells, MYCN binds to the *FAK* promoter, which leads to its overexpression [121]. Moreover, *FAK* downregulation through siRNA decreased cell viability, induced apoptosis, and reduced migration and invasion in *MYCN*-amplified NB cell lines [121,122]. These findings pave the way to study the therapeutic potential of FAK inhibition for the treatment of NB. Neferine, a bisbenzylisoquinoline alkaloid and FAK inhibitor, induces cell cycle arrest in the G2/M phase, autophagy, and apoptosis, and decreases cell migration in vitro [123]. Other small molecule inhibitors such as PF-573,228 and 1,2,4,5-benzenetetraamine tetrahydrochloride (Y15) bind to the autophosphorylation site of FAK, blocking its activity. These inhibitors decreased cell survival, proliferation, invasion, and migration in NB patient-derived xenografts [124]. Another strategy is to use thermal ablation. This approach was tested on a tumor sphere model of NB and denatured collagen type I and IV, which reduced stiffness, cell migration, and mesenchymal transition. Interestingly, collagen denaturation was associated with the decreased phosphorylation of FAK [125]. These studies demonstrate the antitumor effects of reduced FAK activity and demonstrate the potential to target the NB microenvironment.

Other approaches that disrupt the interactions between NB cells and the ECM focus on integrins and adhesion molecules. The expression of αvβ3 integrin in NB cell lines is correlated with increased migration and metastasis, which makes it a potential target for NB treatment [126]. Triazole tetraiodothyroacetic acid (TAT), an antagonist of αvβ3 integrin, has been used in conjugation with benzylguanidine, a norepinephrine transporter inhibitor, to inhibit αvβ3 activity, which reduced tumor growth and metastasis in NB xenografts [127,128]. The inhibition of neural cell adhesion molecule 1 (NCAM or CD56) is under investigation, given its high expression levels in NB [129,130]. Lorvotuzumab mertasine is an antibody–drug conjugate that targets NCAM. It was tested in a phase II clinical trial with relapsed NB. Despite the drug being well tolerated in patients, it did not show sufficient clinical efficacy [131].

Changes in the TME with antitumor effects can also be made by targeting angiogenic processes in NB, since angiogenesis and the overexpression of proangiogenic factors like VEGF are correlated with high-risk NB [111]. Anlotinib is a small molecule tyrosine kinase inhibitor that has anti-angiogenic activity through inhibiting VEGF, fibroblast growth factor receptor (FGFR), and platelet-derived growth factor receptor (PDGFR). Anlotinib decreased the formation of abnormal and leaky blood vessels, reduced the levels of hypoxia, and increased vascular perfusion, which led to high levels of T cell infiltration in NB mouse models [132]. The antitumor activity demonstrated in this preclinical study prompted the onset of a phase II clinical trial to determine the effectiveness and toxicity of anlotinib in combination with other anticancer drugs: irinotecan and temozolomide for the treatment of refractory or relapsed NB (NCT04842526). Bevacizumab, another VEGF inhibitor, was tested in a phase II clinical trial in combination with irinotecan, temozolomide, or topotecan–temozolomide. The addition of bevacizumab to the chemotherapeutic drugs increased the overall response rate, with limited toxicity in relapsed and refractory NB [133]. In summary, targeting angiogenesis represents a promising approach in NB treatment.

## 5. Targeting Immunological Alterations in NB

The dysregulated TME in NB fosters an immunosuppressive phenotype combined with the low expression of MHC-I in NB cells. It also correlates with elevated levels of immune checkpoint proteins and the reduced secretion of pro-inflammatory cytokines, which collectively reduce immune cell activation and compromise effective immune surveillance against NB [134,135]. *MYCN* contributes to this immunosuppressive state by reducing interferon and pro-inflammatory pathways, which diminishes immune activation and limits cytotoxic T cell infiltration [136,137]. Moreover, *MYCN* amplification is correlated with low expression levels of MHC-I, MICA, ULBPs, and PVR, which are ligands for the activating receptors NKG2D and DNAM1 in NK cells, thus impairing T and NK cell-mediated antitumor responses and decreasing the recruitment of cytotoxic T cells and NK cells [138,139]. Conversely, infiltrating T cells are correlated with improved survival rates in NB, highlighting the therapeutic potential of restoring immune function [140,141]. In addition, *MYCN*-amplified tumors also exhibit a downregulated expression of *CCL2*, resulting in the impaired recruitment of monocytes and dendritic cells [142]. *MYCN* further promotes immune evasion by inducing infiltrating immunosuppressive cells, including T regulatory cells, through chemokine-like factor upregulation, tumor-associated macrophages, and cancer-associated fibroblasts [143,144]. ALK can contribute to these immunosuppressive effects as it is involved in *MYCN* upregulation and MYCN protein stabilization, and has been associated with the overexpression of the immune checkpoint PD-L1 [145,146]. Therefore, the approaches discussed in Section 2.2 and Section 3.5, focusing on targeting MYCN and ALK, may be used not only to target tumor growth, but to alleviate immune evasion mechanisms, and their effect could be further potentiated by combining them with immunotherapeutic approaches.

Tumor-associated macrophages (TAMs) and cancer-associated fibroblasts (CAFs) in the TME are also key regulators of immunosuppression. These cells contribute to the secretion of immunomodulatory molecules, mainly TGF-β, which suppresses the cytotoxic effect of CD8^+^ T cells by inhibiting the expression of perforin, granzyme B and A, IFN-γ, and FAS ligand (FASL) [147,148]. Also, it downregulates the expression of activating receptor NKG2D on NK cells, which inhibits their immune activity [149]. In addition, these immunosuppressive cells secrete IL-6 and promote the expression of this cytokine by NB cells, resulting in decreased IL-2-mediated NK cytotoxicity and further enhancing TAM survival [150]. Moreover, both TAMs and CAFs express immune checkpoint ligands for PD-L1 and CTLA-4, inhibiting the activity of effector T cells [151,152]. This immunosuppressive environment not only limits the homing of immune cells, but also promotes their exhaustion. Therefore, targeting TAM and CAF immunomodulatory functions may be essential to enhance the efficacy of immunotherapeutic strategies in NB, including cell-based and antibody-dependent cellular cytotoxicity.

**Figure 4:**
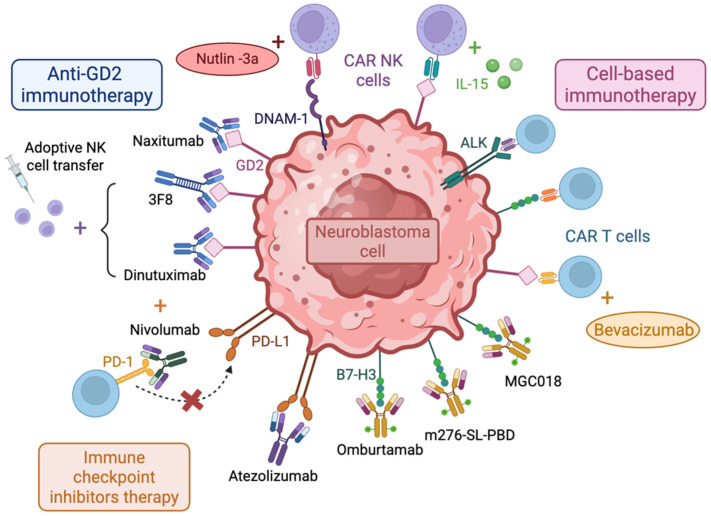
Immunotherapy approaches to treating- NB. GD2 is expressed by NB cells but not by healthy cells, making it an ideal therapeutic target. The FDA has approved three anti-GD2 monoclonal antibodies for NB treatment: naxitumab, 3F8, and dinutuximab. Recent research combines the latter two drugs with adoptive NK cell transfer to enhance efficacy. Immune checkpoint therapies mainly focus on disrupting PD-1/PD-L1 interactions, with current research focusing on nivolumab (anti PD-1) and atezolizumab (anti PD-L1). Moreover, ongoing clinical trials are testing nivolumab with dinutuximab. Another immune checkpoint target, B7-H3, is being explored with novel agents like omburtamab, m276-SL-PBD, and MGC018. Novel immunotherapy approaches focus on cell-based immunotherapy, particularly with CAR immune cells. Anti-ALK, anti-GD2, and anti-B7-H3 CAR T cells have shown promise in preclinical models. Anti-GD2 CAR T cells have also been studied in combination with the VEGF inhibitor bevacizumab to improve outcomes. Anti-DNAM-1 and anti-GD2 CAR NK cells are also under study with Nutlin-3a and IL-15, respectively. These approaches highlight the great variety of targets for immunotherapy and the potential of combining immunotherapies for more effective NB treatment. Created with BioRender.com.

NB cells also secrete arginase-2, an enzyme that catalyzes the hydrolysis of arginine, which is an essential amino acid for T cells. Therefore, by reducing the levels of arginine in the TME, arginase-2 secretion inhibits T cell activation and proliferation, and contributes to the immunosuppressive effect of TME [153]. Altogether, these studies highlight the tremendous changes occurring in the immune ecosystem, suggesting therapeutic opportunities to tackle NB. Understanding the interplay between NB and the immune system paves the way for the development of targeted and effective immunotherapies that have the potential to improve the outcomes of high-risk NB. Novel immunotherapeutic strategies for NB treatment are summarized in Figure 4.

### 5.1. Targeting GD2 as an Approved Immunotherapeutic Treatment of NB

The approval of anti-GD2 antibodies validates the potential of immunotherapy for the treatment of NB. Ganglioside GD2 has limited expression in normal tissue, being expressed only in neurons, peripheral pain fibers, skin melanocytes, and mesenchymal stem cells [154]. It is the most expressed ganglioside in NB cells [155]. Due to the differential expression between malignant and normal cells, GD2 is a key target in NB immunotherapy. In addition, NB cells also secrete soluble GD2, which exerts immunomodulatory effects like inhibiting T cell proliferation [156].

Historically, 3F8, a murine IgG3 monoclonal antibody, was the first anti-GD2 studied for the treatment of NB. It triggers complement activation and induces antibody-dependent cytotoxicity [157,158]. In a phase I clinical trial, only one out of sixteen NB patients had a complete response [159,160]. The lack of effectiveness was associated with the human anti-mouse antibody responses after anti-GD2 treatment, which correlated with reduced persistence in the blood [157]. As a result, naxitumab, the humanized IgG1 form of the 3F8 antibody, was developed. Naxitumab was more effective than murine 3F8 in xenograft NB models, exhibiting a more potent antibody-dependent, cell-mediated cytotoxicity and reduced complement-mediated cytotoxicity [161]. When tested in combination with granulocyte macrophage colony-stimulating factor (GM-CSF) in a phase I clinical trial, it showed minimum toxic effects, low immunogenicity, and sufficient anti-NB activity [162]. Then, in a phase II clinical trial in high-risk NB patients, naxitumab produced antitumor efficacy, with 59% of patients exhibiting a complete response [163]. Considering the success of naxitumab in clinical trials, the FDA approved its use in combination with GM-CSF for high-risk NB patients that have shown either a partial response, minor response, or a stable disease response to previous therapy. Despite its approval, treatment with naxitumab can lead to symptoms such as severe pain and hypotension, requiring the close monitoring of patients [157].

Dinutuximab is a human/mouse chimeric antibody targeting GD2, whose mechanism of action also depends on antibody-dependent cellular cytotoxicity. In a phase III trial, dinutuximab was administered in combination with GM-CSF and IL-2 to high-risk NB patients that had responded to induction and consolidation therapy. Dinutuximab improved overall survival, which granted its FDA approval [164]. However, severe adverse effects have been reported following dinutuximab treatment, including immunodeficiency, thrombocytopenia, urticaria, and anemia [165]. An analog of this antibody, dinutuximab beta, is also in the market for the treatment of NB, and showed antitumor activity and similar toxicity profiles [166]. This antibody is commercialized under the named Qarziba^®^, and it is indicated for patients older than 12 months who have shown a response to previous treatments. In a recent phase II study performed in relapsed/refractory high-risk NB patients, dinutuximab beta was tested in combination with chemotherapy (temozolomide or temozolomide–topotecan), and it increased overall response rate and progression free survival compared to chemotherapy alone [167]. Further studies need to be carried out to reduce toxicities while maintaining clinical efficacy. Although anti-GD2 antibodies have improved the treatment of high-risk NB patients, still almost half of them relapse. Resistance mechanisms were associated with reduced GD2 expression, which correlated with the mesenchymal cell state of NB cells. Pharmacological inhibition with the FDA-approved EZH2 inhibitor, tazemetostat, de-repressed GD2 expression and sensitized NB cells to anti-GD2 antibodies. These results support the combination of EZH2 inhibitors and anti-GD2 antibodies to enhance outcomes for NB patients [168].

### 5.2. Immune Checkpoint Inhibitor (ICI) Therapy

Immune checkpoints are cell membrane proteins that inhibit cytotoxic lymphocyte activity after antigen recognition to protect normal tissue. These proteins are often upregulated in NB, allowing NB to escape from immune cell attacks. Programmed death-ligand 1 (PD-L1) is a protein from the B7 family of immune regulatory molecules that can be expressed on tumor cells. Upon the binding of its receptor (PD-1) on T or NK cells, it inhibits immune responses [135]. Several studies have associated high PD-L1 expression with a decrease in overall survival in NB [169,170,171].

Nivolumab is a humanized Ig-4 monoclonal anti-PD-1 antibody. It is approved by the FDA for the treatment of melanoma, non-small cell lung cancer, renal cell carcinoma, Hodgkin lymphoma, colorectal cancer, and hepatocellular carcinoma. Recently, nivolumab was tested in a phase 1–2 clinical trial in monotherapy for NB patients. No objective response was achieved, although 50% of the evaluated patients showed stable disease after treatment [172]. Atezolizumab is a humanized anti-PD-L1 antibody, recently approved by the FDA for the treatment of non-small cell lung cancer. In a phase 1–2 study in NB patients, atezolizumab monotherapy produced a progression-free survival of 2.6 months [173]. Thus, these clinical studies indicate that monotherapy targeting PD-1/PD-L1 interactions is not sufficiently effective against NB, as it does not produce enough antitumor activity to induce a response. However, these immunotherapies reduced disease progression, indicating that combining this type of therapy with other agents may help generate an effective anticancer response.

Chemotherapy agents have demonstrated immunostimulatory effects, mediating immunogenic cytotoxicity, the release of tumor antigens, and immune cell trafficking [174]. The efficacy of the antitumor response achieved with the combination of chemotherapy and ICIs has granted it FDA approval for several cancers, including metastatic non-small cell lung cancer [175] and metastatic small cell lung cancer [176], supporting the rationale for similar strategies for high-risk NB. However, no objective response was observed in NB patients when nivolumab was tested in combination with the chemotherapeutic agent cyclophosphamide in a phase II clinical trial [177]. Interestingly, a case report illustrated that the administration of nivolumab and dinutuximab beta led to objective responses in two patients, with one achieving complete remission and the other experiencing a partial response [178]. Radiotherapy is also a good candidate for achieving a synergistic effect with ICIs, as it has immunomodulatory functions through the abscopal effect, inducing the secretion of inflammatory cytokines and chemokines in the TME, the release of new tumor antigens from apoptotic cancer cells, and the migration of effector T cells to enhance local and distant tumor control [179]. Recently, radiotherapy in combination with ICI was approved by the FDA for cervical cancer [180], paving the way for similar strategies to be explored in NB. Currently, there is an ongoing phase 1 clinical trial (MiNivAN), which aims to investigate the combination of radiotherapy ^131^I-MIBG, dinutuximab, and nivolumab, to provide further insights into their potential synergistic effects against NB (NCT02914405).

Novel combination strategies to increase NB susceptibility to PD-1 therapy have explored the genetic silencing of ERAP1, an enzyme that processes peptides for presentation on MHC-I molecules, through CRISPR/Cas9 and the HDAC inhibitor entinostat [181]. This study showed that entinostat increased MHC-I and PD-L1 expression in murine NB cell lines, while ERAP1 inhibition enhanced the activation of effector T cells and NK cells, most likely through increased neo-antigen presentation on cancer cells. These effects, combined with the PD-1 blockade, resulted in increased antitumor responses and reduced tumor growth. This elucidates the alternative of using epigenetic and genetic techniques to potentiate ICI antitumor effects in NB; further research is needed to validate these findings in clinical settings, particularly given the current limitations associated with CRISPR/Cas9 in terms of safety and delivery [182].

B7-H3, another immune checkpoint from the B7 protein family, shows immunosuppressive action, inhibits NK cell recognition and cytotoxic responses, and suppresses the production of IFN-γ, IL-2, IL-10, and IL-13, resulting in T cell inactivity [183,184,185]. In NB, B7-H3 expression is correlated with poor event-free survival [186]. In patient-derived and cell line-derived xenografts, targeting B7-H3 with the pyrrolobenzodiazepine-armed antibody–drug conjugate m276-SL-PBD prolonged event-free survival in mice NB models [187]. Moreover, Vobramitamab duocarmazine (also known as MGC018) is an investigational duocarmycin-based antibody–drug conjugate directed against the B7-H3 antigen, which has shown success in preclinical studies. In one study, it induced an objective response in three out of nine of the mice models tested, and two showed stable disease after treatment [188]. In addition, in a more recent study, it showed cytotoxic activity specifically against cell lines expressing human B7-H3, and reduced tumor growth and increased survival in NB mice models [189].

Moreover, by using single-cell RNA-sequencing to elucidate immunoregulatory interactions in NB, the binding between NECTIN2 (on the NB surface) and TIGIT (on the immune cell membrane) was identified as a crucial immune checkpoint, which inactivates T and NK cells in the TME [190]. Blocking TIGIT increased T and NK cells’ antitumor immunity. This study highlights the potential of exploring therapeutic approaches for immune checkpoint inhibitors in NB, aiming not only to improve the targeting of extensively studied immune checkpoints, but also to investigate novel targets. Overall, while significant advancements have been achieved in immune checkpoint therapies, continued research is necessary to enhance their therapeutic efficacy in patients.

### 5.3. T Cell and CART Approaches in NB

T cells depend on MHC-I recognition to detect their targets, which is crucial for initiating cytotoxic responses against tumor cells and inducing effective antitumor immune responses. In NB cells, the nuclear factor kappa B (NFκB) signaling pathway promotes an undifferentiated phenotype and also downregulates expression of MHC-I [191,192]. Interestingly, *MYCN* overexpression has been correlated with reduced MHC-I levels, although a direct regulatory effect has not been established [192]. Low expression of MHC-I in NB correlates with poor clinical outcomes [193,194]. This is consistent with the effects of MHC-I expression on the density of tumor-infiltrating lymphocytes, with a higher expression of the antigen associated with a higher density of T cells, which is an indicator of better prognosis [141,195]. Thus, the downregulation of MHC-I expression impacts the effectiveness of T cell-mediated cytotoxic responses against tumor cells, and strategies to restore its expression are of therapeutic interest. The class 1 HDAC inhibitor, entinostat, has been shown to upregulate MHC-I in NB, resulting in the enhanced cytotoxicity of tumor-specific T cells, and it also increased the expression of MICA/MICB, augmenting NK recognition of tumor cells as well [196]. This highlights a potential synergistic effect between epigenetic modulation and cell-based immunotherapies, but its applicability in clinical settings remains to be explored.

Given the limitations associated with MHC-I downregulation in tumors, alternative strategies that avoid MHC-I dependence are of great interest. CAR T cells have revolutionized cancer therapy, offering a promising immunotherapeutic strategy that allows for the recognition of tumor antigens independent of MHC-I. While they have demonstrated efficacy in acute lymphoblastic leukemia, their application in solid tumors represents a more difficult challenge. Recent efforts have been made to improve the efficacy of CAR T cells in solid tumors. Straathof et al. engineered GD2-targeting CAR T cells, featuring CD28 and CD3ζ signaling domains, and integrating a humanized single-chain fragment variable (scFv) against GD2. In a phase 1 clinical trial with relapsed/refractory NB patients, the treatment was well tolerated and led to immune activation following treatment. However, patients did not achieve an objective response [197]. Yu et al. developed other CAR T cells, with an anti-GD2 scFv domain, CD3ζ, CD28, and 4-1BB signaling domains, and an inducible caspase 9 domain, for the potential elimination of the CAR T cells in the case of adverse effects. The phase 1 study showed that the treatment was safe, and that the side effects like neuropathic pain and cytokine release syndrome were mild. Four out of twelve patients showed long-term stable disease, although no objective response was reached [198]. Similarly, Del Bufalo et al. studied the efficacy of GD2-CAR T cells armed with CD28, 4-1BB, and inducible caspase 9, in a phase 1–2 clinical trial in patients with relapsed or refractory high-risk NB. In this trial, 63% of the patients showed a response to the treatment, while 74% of the patients experienced cytokine release syndrome [199].

The limitation of GD2-CAR T cell therapy in NB may be due to cell infiltration in tumor sites. One approach gaining attention involves combining GD2-targeting CAR T cells with chemotherapy and immune checkpoint therapy. A clinical trial (NCT01822652) tested a treatment with third-generation GD2-CAR T cells, alone or in combination with cyclophosphamide, fludarabine, or GD2-CAR T cells with cyclophosphamide, fludarabine, and pembrolizumab (PD-1 inhibitor). Although the treatment was well tolerated, the combined treatment produced only modest responses, indicating the need for further optimization [200]. Strategies to improve efficacy may include selecting patients based on the expression of immune-related biomarkers, and while predictive biomarkers for ICI response have not yet been established in NB, previous research in other solid tumors suggests that tumor mutational burden and PD-L1 expression may serve as reliable indicators [201]. Biomarker-guided combination regimens may help to identify patient subgroups, most likely to benefit from dual CAR T and ICI therapy.

Bocca et al. studied the combination of GD2-CAR T cells and bevacizumab, a humanized antibody directed towards VEGF-A in an orthotopic xenograft model of human NB. The rationale is that neo-angiogenesis generates an immunosuppressive microenvironment, and that bevacizumab may reprogram tumor vasculature. With bevacizumab, GD2-CAR T cells infiltrated the tumor significantly more, which induced antitumor activity. Interestingly, GD2-CAR T cells also produced interferon-γ (IFN-γ), which upregulated PD-L1 in NB cells, supporting the addition of the PD-L1 blocker to the combination [202].

Another potential target for CAR T cell immunotherapy against NB is the checkpoint molecule B7-H3. Du et al. developed B7-H3 CAR T cells and reported that B7-H3 CAR T cells limit tumor growth and prolonged overall survival without detectable toxicity [203]. Moreover, Moghini et al. engineered a novel B7-H3 CAR T cell using the synthetic Notch strategy. These CAR T cells are designed to recognize the GD2 antigen, which then allows them to recognize B7-H3, and this two-step process ultimately leads to the activation of the CAR T cells. These novel T cells were resistant to exhaustion, showed antitumor efficacy, and did not cause neurotoxic effects in xenograft models of metastatic NB [204]. Moreover, immunotherapy targeting ALK is another approach for NB treatment. Walker et al. designed CAR T cells directed against ALK. However, the low density of ALK in NB cells limited the activity of these CAR T cells in mice [205]. The generation of novel CAR T cells able to integrate ALK CAR T cells with chimeric co-stimulatory receptors targeting B7-H3 or GD2 proved the antitumor efficacy of anti-ALK CAR T cells [206].

### 5.4. NK Cells and CAR NK Approaches in NB Treatment

While CAR T cells have shown promise, they have been associated with severe adverse effects. Cell-associated neurotoxicity syndrome and cytokine release syndrome are the main reasons for hospitalization following CAR T cell treatments. Interestingly, NK cells after activation have a different cytokine release profile than CAR T cells, and therefore do not produce the side effects commonly seen after CAR T cell treatment [207]. As NB cells can escape recognition by T cells, they can also develop mechanisms to avoid NK cell antitumor activity, by reducing the ligand expression of NK cell-activating receptors [156,208]. For instance, NB cells express low levels of MICA/B and ULBPs, which are recognized by NKG2D receptors in NK cells, as well as PVR and Nectin-2 ligands, which are recognized by DNAM1 receptors in NK cells [139,209].

Harnessing the immunosuppressive characteristics of NB cells and their evasive mechanisms against NK cell attack represents a promising avenue in immunotherapy. Importantly, NK cell-based therapy is an allogeneic adoptive transfer, in which NK cells from an HLA-matched or haploidentical donor are isolated, expanded, and activated before the transfer [210].

In order to increase the antitumor effect of adoptive NK cell transfer, a combination with dinutuximab was achieved in immunodeficient mice, which demonstrates a reduction in metastasis and increased survival [211]. The combination of dinutuximab, N-803 (IL-15 superagonist), and adoptive NK cell transfer increased NK cell-mediated cytotoxicity in vitro by stimulating the antibody-dependent cellular cytotoxicity of NK cells, and increased the survival of NB xenograft models [212]. Moreover, in a phase 1 study, adoptive NK cell transfer was combined with anti-GD2 murine monoclonal antibody 3F8 in 35 NB patients who had received chemotherapy prior to the study. The results indicated that 29% of patients achieved a complete or partial response [213]. Adoptive NK transfer was studied in combination with other agents. Veneziani et al. combined adoptive NK cell transfer with Nutlin-3a, an MDM2 antagonist. MDM2 is a protein known for its role as a negative regulator of the tumor suppressor *p53*. Treatment with Nutlin-3a increased the expression of PVR and Nectin-2, increasing NK cell-mediated cytotoxicity and improving mice survival [214].

Another novel NK cell-based immunotherapy is the use of CAR NK cells, combining the innate cytotoxicity of NK cells with the specificity of CAR. Thus, NK CAR cells offer a promising alternative to effectively combat NB. Focacceti et al. developed CAR NK cells expressing DNAM-1 fused with a CD3ζ stimulatory domain. They showed that these CAR NK cells showed an increase in degranulation, increased the production of cytokines IFNγ and TNFα, and showed stronger cytotoxic activity compared to NK cells. Interestingly, previous treatment of NB cells with Nutlin-3a enhanced the degranulation of DNAM-1-CD3ζ CAR NK cells, increasing the susceptibility of NB cells to CAR NK cells [215]. In another study, Bodden et al. engineered NK cells to co-express GD2-CAR and an IL-15 superagonist. The authors demonstrated that these cells exhibit selective cytotoxicity against a GD2-expressing NB cell line. Additionally, they observed an enhanced cytotoxic effect resulting from the combined action of both natural and CAR-mediated NK cell mechanisms [216]. Recently, Chu et al. demonstrated the cytotoxic activity of anti-ROR1 CAR NK cells in combination with an IL-15 superagonist (N-803) against NB cell lines and the ability of this therapy to induce tumor control and enhance survival in NB mice models. Interestingly, they also found that N-803 increased CXCR3 expression on engineered cells, a chemokine receptor that mediates tumor homing [217]. Thus, this study proposes a strategy to improve tumor trafficking and the infiltration of CAR NK cells, which is a major challenge in cellular immunotherapies against solid tumors. Other approaches that have been explored in solid tumors include engineering NK cells to express chemokine receptors that facilitate tumor homing—this could also be explored in future research on CAR NK cells to improve their effectivity against NB [218]. Overall, these results collectively provide strong evidence for the potential of CAR NK cells as a viable therapeutic approach in combating NB, paving the way for further in vivo studies, and offering hope for improved treatment outcomes in the future.

## 6. Future Perspectives

Neuroblastoma remains a clinically challenging cancer with high heterogeneity and poor outcomes in high-risk patients. The heterogeneity and plasticity between adrenergic and mesenchymal phenotypes in NB contribute to the variability in clinical outcomes and present significant challenges in translating preclinical findings into effective clinical treatments. Although advanced single-cell approaches have provided new insights into the genetic and epigenetic landscapes of NB, highlighting novel therapeutic targets and pathways, further studies are needed to design therapies that effectively target this tumor. Future research should focus on establishing reliable biomarkers for personalized therapy design and the robust prediction of clinical outcomes. Moreover, therapies should be rationally designed to combine genetics-, epigenetics-, TME-, and immunotherapy-based strategies to enhance their efficacy as monotherapies and overcome the barriers imposed by the multiple hallmarks of NB. To achieve this, a collaborative approach is needed, leveraging a large number of patient samples to account for NB’s heterogeneity and improve both our understanding of NB and the translational value of preclinical research.

## 7. Concluding Remarks

Significant efforts have been directed towards improving the survival rates of high-risk NB patients while reducing the adverse effects of current treatments. Novel strategies to target genetic and epigenetic alterations in NB tumors include the use of PROTACs, which offer an alternative to traditional small molecule inhibitors or to targeting gene mutations directly with PIPs conjugated with DNA alkylating agents. Another promising approach involves targeting immune alterations within NB through immune checkpoint inhibitors and cellular therapies, such as CAR T and CAR NK cell-based therapies. In addition, new therapeutic combinations of both types of immunotherapies and the incorporation of chemotherapeutic and epigenetic drugs into immunotherapies are being studied to increase efficacy and minimize the side effects compared with monotherapies. In particular, epigenetic interventions may sensitize NB cells to treatment and reduce the required doses of chemotherapy, thereby decreasing toxicity. Similarly, immunotherapies offer more targeted tumor cell elimination with fewer systemic side effects. These approaches, alone or in combination, are a promising avenue for mitigating the adverse effects associated with conventional therapy. Ongoing efforts to integrate targeted therapies, immunomodulation, and epigenetic reprogramming hold great promise for transforming NB treatment.
